# Regarding: the accuracy of the Candida Score^®^ in predicting the likelihood of fungal sepsis in newborns

**DOI:** 10.1080/07853890.2025.2578732

**Published:** 2025-10-31

**Authors:** Zainab Hafeez

**Affiliations:** Nishtar Medical University and Hospital, Multan, Pakistan

**Keywords:** Candida Score, Neonatal Sepsis, Fungal Infection

It was a pleasure to read with great interest the recent article by Siswanto et al. on the revised Candida Score for early detection of neonatal candidemia [1]. The study provides valuable insights into risk stratification of fungal sepsis in neonates, yet I would like to highlight several points that merit further discussion.

First, the revised Candida Score was not evaluated against established fungal biomarkers, such as serum (1,3)-B-D-glucan or mannan antigen testing [2]. These assays, while not universally available, are increasingly used as adjunctive diagnostic tools for invasive candidiasis and are endorsed in many international guidelines. Without a direct comparison, it is difficult to determine whether the revised score offers superior, equivalent, or complementary diagnostic value. Even if logistical or economic barriers preclude routine biomarker testing in certain settings, reporting such comparisons would allow readers to better contextualize the model within existing diagnostic pathways and to assess its generalizability beyond the study site.

Second, although the study reports a strikingly high mortality rate of 42% among neonates with candidemia, the analysis did not explore whether earlier initiation of antifungal therapy, prompted by the revised Candida Score, might have reduced mortality. The true utility of any predictive score extends beyond diagnostic accuracy; its impact on clinical outcomes such as survival, length of hospitalization, and antifungal stewardship is equally critical [3]. A sensitivity analysis or discussion of how score-guided interventions might alter outcomes would have strengthened the clinical relevance of the findings.

Third, while the authors briefly noted the distribution of Candida albicans and non-albicans species, the implications of species-specific epidemiology were not explored in depth. This is particularly important because non-albicans Candida (e.g. C. parapsilosis, C. glabrata) frequently display differences in virulence, antifungal susceptibility, and clinical prognosis compared with C. albicans [4]. Failure to account for these differences may obscure key variations in risk factors and treatment responses. Stratified analyses by species could help clinicians understand whether the revised score performs equally well across different Candida species, or whether modifications are needed for certain subgroups.

In conclusion, while the revised Candida Score represents a promising approach to the early identification of neonatal candidemia, further studies incorporating biomarker comparisons, outcome analyses, and species-specific data are warranted. These steps would enhance the quality, external validity, and clinical utility of the model.

## Data Availability

Data sharing is not applicable to this article as no data were created or analysed in this study.

## References

[1] Siswanto JE, Patriani D, Boromeus AD, et al. The accuracy of the Candida Score^®^ in predicting the likelihood of fungal sepsis in newborns. Ann Med. 2025;57(1):2548022. doi: 10.1080/07853890.2025.2548022. 40844406 PMC12377106

[2] Shabaan AE, Elbaz LM, El-Emshaty WM, et al. Role of serum (1,3)-B-d-glucan assay in early diagnosis of invasive fungal infections in a neonatal intensive care unit. J Pediatr (Rio J). 2018;94(5):559–565. doi: 10.1016/j.jped.2017.07.020. 29144965

[3] Cahan H, Deville JG. Outcomes of neonatal candidiasis: the impact of delayed initiation of antifungal therapy. Int J Pediatr. 2011;2011:813871–813876. Epub 2011 Nov 3. PMID: 22121380; PMCID: PMC3216279. doi: 10.1155/2011/813871. 22121380 PMC3216279

[4] Silva S, Negri M, Henriques M, et al. Candida glabrata, Candida parapsilosis and Candida tropicalis: biology, epidemiology, pathogenicity and antifungal resistance. FEMS Microbiol Rev. 2012;36(2):288–305. doi: 10.1111/j.1574-6976.2011.00278.x. 21569057

